# Imaging for illuminating actionable pathways in breast cancer

**DOI:** 10.1097/CCO.0000000000000908

**Published:** 2022-09-12

**Authors:** Laura Gilardi, Lighea Simona Airò Farulla, Francesco Ceci

**Affiliations:** a Division of Nuclear Medicine, IEO European Institute of Oncology IRCCS; b Department of Oncology and Hemato-Oncology, University of Milan, Milan, Italy

**Keywords:** inhibitors of the fibroblast activation protein, inhibitors of the fibroblast activation protein breast cancer, 2-deoxy-2-[^18^F]-fluoro-d-glucose, 2-deoxy-2-[^18^F]-fluoro-d-glucose breast cancer, FES, FES breast cancer, positron emission tomography

## Abstract

**Purpose of review:**

Nuclear medicine has the potential to explore and illuminate several pathways in breast cancer (BC) offering different radiopharmaceuticals for positron emission tomography (PET) designed to target specific tumor characteristics. The aim of this critical review is to give an overview about emerging opportunities in PET imaging, underlining the future potential contribution in the management of BC patients.

**Recent findings:**

Beside 2-deoxy-2-[^18^F]-fluoro-d-glucose (FDG), new generation tracers for PET imaging have been recently proposed to investigate specific characteristics in breast cancer, both targeting tumor cells and the tumor micro-environment (TME).

**Summary:**

FDG-PET is a procedure that received extensive clinical validation. However, its role in BC is still suboptimal due to the low-FDG avidity of specific tumor subtypes. Human epidermal growth receptor-2 and integrin targeted PET radiotracers might provide useful information selecting patients more likely to respond to target therapy. FluoroEstradiol (FES) is a FDA-approved PET radiotracer targeting the estrogen receptor (ER), useful to investigate metastatic ER+ patients, to assess in vivo ER heterogeneity and to evaluate hormonal therapy efficacy. Inhibitors of the fibroblast activation protein (FAPi) targeting the cancer-associated fibroblast can explore the TME with PET imaging. FAPi is also proposed a theranostic agent for radio-ligand therapy.

## INTRODUCTION

Breast cancer (BC) is the most common cancer among women worldwide [1] and, even if the chance of cure is good, the prognosis worsens dramatically in case metastatic BC [2]. The introduction of mammography as screening procedure improved the overall mortality rate over the past years [1]. However, new generation imaging might positively impact the management of BC patients, as already happened in other malignancies. The initial stage of the disease and the evaluation of response to systemic therapy in case of metastatic BC (MBC) cannot rely anymore on computed tomography (CT) and bone scintigraphy, only [2]. Positron emission tomography (PET) is a functional imaging technique able to ‘illuminate’ specific cancer related pathways [2]. 

**Box 1:**
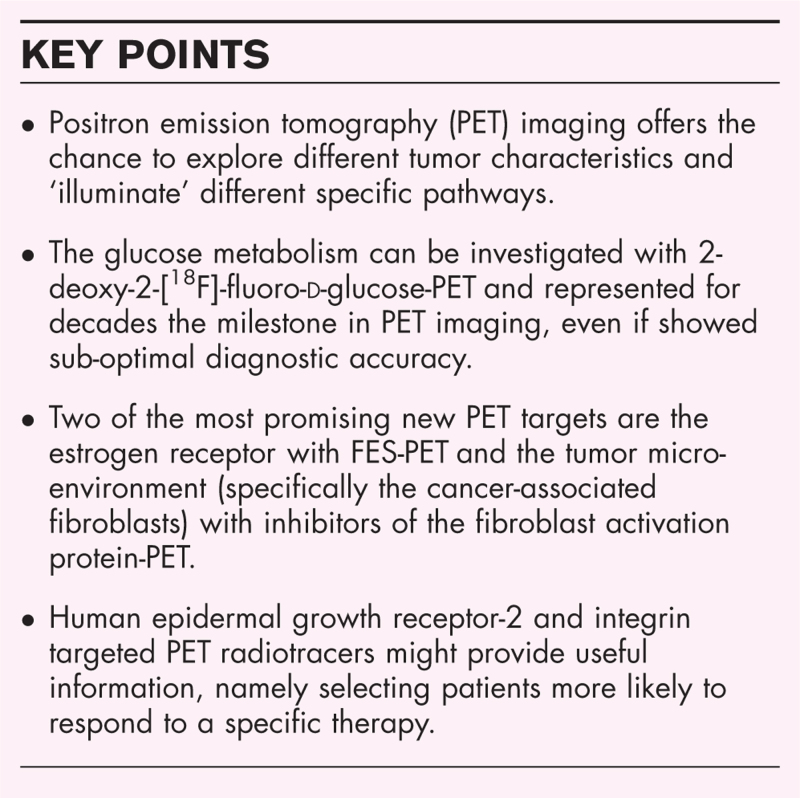
no caption available

## GLUCOSE METABOLISM TARGETED POSITRON EMISSION TOMOGRAPHY RADIOPHARMACEUTICAL

**FIGURE 1:**
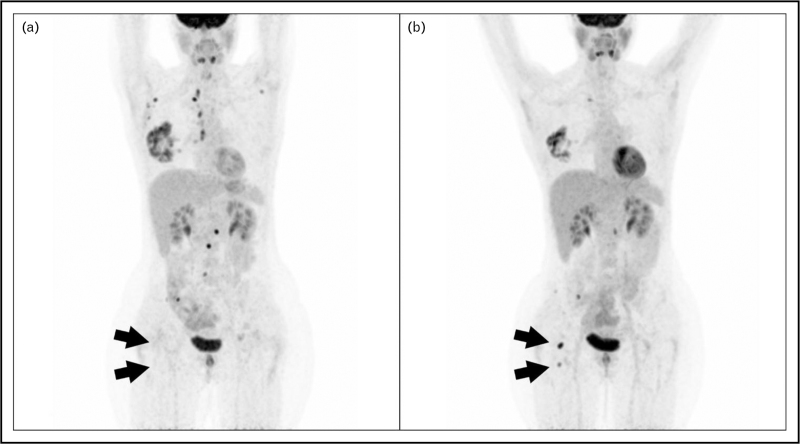
41-year-old female patient with metastatic (lymph node, lung, and skeletal localizations) invasive ductal breast carcinoma (Estrogen Receptor (ER) 95%, Progesterone Receptor (PR) 5%, HER2 0, Ki-67 50%), previously treated with Denosumab, Ribociclib, and Letrozole and with radiotherapy to the right femoral head and iliac bones. The patient underwent FDG-PET to restage the disease. FDG-PET (a) showed complete response to radiotherapy of the right femoral head lesion (arrows), with other lesions remained stable (SD). Subsequently capecitabine was delivered and the patients underwent a second scan four months later, to evaluate the response to therapy. FDG-PET (b) showed a partial response of the primary right mammary lesion, bilateral axillary and mediastinal lymph nodes, and skeletal lesions at the lumbar spine. However, new hyper-metabolic lesions appeared it the right femoral head (arrows) and, overall, the scan has been interpreted as progressive disease (PD). ER, estrogen receptor; FDG-PET, positron emission tomography imaging with 2-deoxy-2-[^18^F]-fluoro-d-glucose; HER2, human epidermal growth receptor-2.

Among different radiopharmaceuticals, PET imaging with 2-deoxy-2-[^18^F]-fluoro-d-glucose (FDG-PET) has been broadly used as diagnostic procedure to investigate BC [3]. FDG-PET offers an ‘in vivo’ overview of the glucose metabolism, and lead to an accurate location of hyper-metabolic sites. According to European Society of Medical Oncology (ESMO) guidelines FDG-PET is recommended in the therapeutic planning of patients with suspected oligometastatic disease as systemic imaging staging, instead of CT and bone scan, due to its higher diagnostic accuracy to correctly detect locoregional and distant site in a one-stop-shop examination [3]. FDG-PET demonstrated a diagnostic accuracy comparable with magnetic resonance imaging (MRI) in detecting bone metastases, which is the current gold standard, outperforming CT and bone scintigraphy [4]. Furthermore, positive FDG-PET can influence the clinical decision making-process of MBC, while a negative scan is highly predictive of a nonmetastatic disease [5^▪^] (Fig. 1).

However, FDG-PET did not fully achieve its initial expectations and, at present, its use in daily practice is still limited to specific clinical settings. One of the main limitations is represented by the limited spatial resolution: due to the partial volume effect the detection of small lesions (<5 mm) is challenging [6^▪^]. The brain physiological FDG uptake limits the exportability of the nervous central system, and thus CT or MRI scan are mandatory to exclude the presence of brain metastasis [6^▪^,^7^]. Nevertheless, the main limitation for FDG-PET is represented by the high heterogeneity of BC on a molecular level. Primary tumors are exposed to various stressful environments, such as oxidative stress, hypoxia, and acidosis, with rapid growth, thus accelerating their heterogeneity also in pathological features, therapeutic response, and prognosis [8]. In the histological classification of BC the invasive ductal and the invasive lobular cancers, holds different FDG uptake, the latter having lower avidity than the former. [9,10]. Even at the molecular level, the identification of subtypes including the estrogen receptor positive subtype (ER+; the 70% of cases of metastatic disease), human epidermal growth receptor-2 overexpressed (HER2+) tumors and triple-negative breast cancer (TNBC) show substantial differences in glucose metabolism [9]. FDG uptake is lower in well differentiated ER+ and in progesterone receptor-positive (PR+) tumors than ER− tumors and in PR− tumors, while TNBCs have higher SUV values than other subtypes [10]. Tumors sub-types with a low Ki-67 (e.g. grade I) also showed lower FDG-avidity compared to grade III [10]. Hence, FDG uptake can be considered as prognostic factor, as the SUVmax (maximum standardized uptake value) is associated with poorer outcome [11]. Nevertheless, in up to 17% of metastatic patients, molecular and genetic profile is different in primary lesion compared to metastatic site.

**FIGURE 2:**
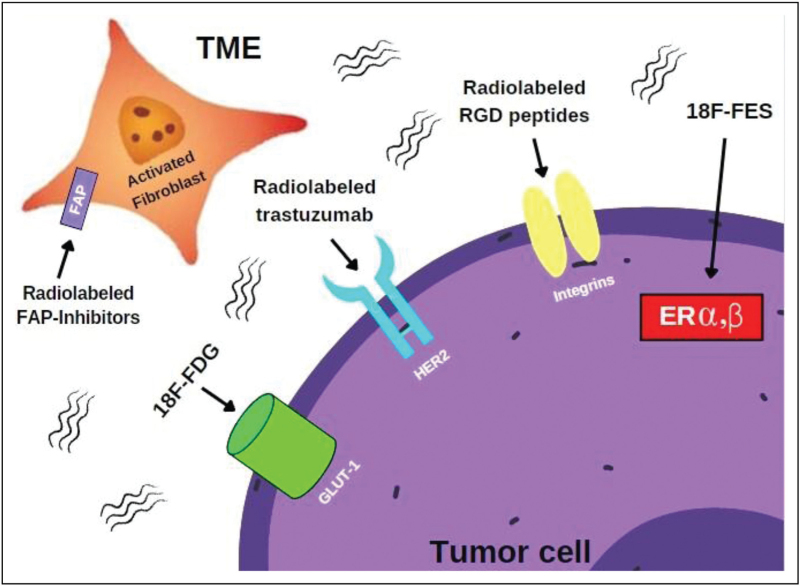
Positron emission tomography (PET) has the potential to illuminate several pathways in breast cancer, as different radiopharmaceuticals have been designed to explore specific tumor characteristics. In this figure are displayed the metabolic and receptorial targets that reached a clinical application. Beside FDG-PET generally applied in clinical practice to evaluate ‘in vivo’ the hyper-activation of glucose metabolism, FES-PET, HER2 and integrin targeted PET have the potential to assess different characteristics of the tumor cell. FAPi-PET provides an ‘in vivo’ overview of the tumor micro-environment, evaluating the expression of the fibroblast activation protein by the cancer associated fibroblasts. FAPi-PET, inhibitors of the fibroblast activation protein positron emission tomography; FDG-PET, positron emission tomography imaging with 2-deoxy-2-[^18^F]-fluoro-d-glucose.

Tumor heterogeneity may result in asynchronous metastases from the primary tumor either as linear or parallel progression, as explained by different models of metastatic evolution [12^▪^]. Accordingly, BC is an extremely heterogeneous disease showing inter- and intra-tumor heterogeneity expressed as both spatial and temporal heterogeneity [13,14]. The methodological approach ‘one-fits-all’, has happened for FDG-PET, should be replaced by a personalized and patient-tailored approach, and further radiopharmaceuticals offer the chance to explore different tumor characteristics and to ‘illuminate’ in vivo with PET imaging different specific pathways, as summarized in Fig. 2 and Table 1.

**Table 1:** Summary of PET radiopharmaceuticals in breast cancer investigated in clinical studies

	Target	Imaging targeting agent	Tumor entity	Characteristics	Limitations
[^18^F]FDG	Glycolysis	Glucose analog	Invasive ductal BC, ER+/− BC, PR+/− BC, TNBC	1. Diagnostic accuracy comparable to MRI 2. Reflects cellular glycolysis 3. Applied in breast cancer staging, re-staging, treatment planning and monitoring	1. Invasive lobular BC, ER+/PR+ BC and BC with low proliferation show low FDG-avidity 2. Central nervous system cannot be explored due to FDG physiological uptake 3. Limited spatial resolution
[^18^F]FES	ER	Estradiol	ER+ BC	FES-PET demonstrated approximately 77% positive agreement and 100% negative agreement compared with ER IHC, and quantitative FES-PET analysis leads to differentiating ER+ from ER− lesions	1. Therapy can interfere with the kinetics and biodistribution of ^18^F-FES 2. Preferential affinity for ERα over ERβ
[^68^Ga]FAPi	FAP (CAFs)	Quinoline FAP inhibitor	FAP+ BC	1. High accuracy in detecting primary breast lesions 2. High tumor to background ratio 3. Can be used as therapeutic agent (radioligand therapy)	Limited clinical evidence currently available
[^89^Zr]Trastuzumab	HER2	Antibody	HER2+ BC	1. Optimal TBR especially in the liver, spleen, kidneys, and brain 2. Tumor lesion SUV^max^ is higher in patients with HER2+ BC than in HER2− 3. Support clinical decision-making when HER2 expression cannot be assessed with biopsy	1. SUV values only helps to differentiate between IHC0 and IHC1–2–3 lesions only 2. Liver and spleen have high uptake in both HER2+ and HER2− BC patients, leading to potential understaging
[^64^Cu]DOTA-Trastuzumab	HER2	Antibody	HER2+ BC	1. Radiation exposure comparable to FDG-PET 2. Differentiation of HER2+ from HER2− BC	1. High liver uptake leading to potential understaging of small lesions 2. Clinical data are still limited
[^18^F]Galacto-RGD	Integrin α_v_β_3_	Amino acids	α_v_β_3_ integrin+ BC	Allow imaging of αvβ3 expression that is correlated with its uptake in PET image	Low sensitivity for lymph node staging
[^89^Zr]Bevacizumab	VEGF	Antibody	VEGF+ BC	1. Higher uptake in tumor lesions than in healthy tissues 2. The replication rate was directly related to the radiotracer uptake	Low sensitivity for lymph node staging

BC, breast cancer; ER, estrogen receptor; FAP, fibroblast activation protein; FDG, 2-deoxy-2-[^18^F]-fluoro-d-glucose; HER2, human epidermal growth receptor-2; IHC, immunohistochemistry; PET, positron emission tomography; VEGF, vascular endothelial growth factor.

## ESTROGEN RECEPTOR TARGETED POSITRON EMISSION TOMOGRAPHY RADIOPHARMACEUTICALS

Hormone-sensitive BC (ER+ or PR+) represents about the 75% of newly diagnosed BC and up to 70% of cases of metastatic disease are estrogen receptor positive (ER+) tumors [1,9]. The gold standard for assessing ER and PR profile is immunohistochemistry (IHC). However, ICH holds some limitations due to sampling errors or the lack of feasibility in performing biopsy of metastatic sites [15]. Moreover, tumor heterogeneity, affects receptor expression on inter- and intra-patient level [15].

In this scenario, a whole-body imaging technique that allows the evaluation, in a single step of ER/PR profile could be a game-changer in the management of patients with hormone-sensitive BC. Among the radiopharmaceuticals designed for ER imaging, the Food and Drug Administration recently approved the use of 16α-[^18^F]17β-estradiol (^18^F-FES) for PET imaging (FES-PET) in metastatic ER+ BC patients, while the approval by the European Medicine Agency is under evaluation. FES has similar affinity as the natural estrogen and binds the ligand binding domain of ER protein of the nucleus, and then accumulate within ER-expressing cells [15]. ^18^F-FES can bind both ER isoforms, with a preferential affinity for ERα over ERβ, while other PET radiopharmaceuticals with higher or exclusively affinity for the ERβ isoform proved limited evidence of efficacy [15–17]. One of the largest prospective studies comparing FES-PET with ER IHC demonstrated approximately 77% positive agreement and 100% negative agreement, concluding that FES-PET is accurate for determining ER expression. Hence, quantitative analysis of PET parameters is feasible to differentiate ER+ from ER− lesions [18^▪^]. These findings have been confirmed by other prospective studies [19,20], designed to evaluate the impact on clinical management in this subset of BC patients (even in stage IV patients) [18^▪^,^19^,^20^,^21^]. Recently, FES-PET has been proposed as prognostic imaging biomarkers for ER-positive MBC patients, as quantitative and qualitative PET parameters were significantly associated with progression-free survival patients receiving endocrine therapy. However, hormonal therapy can interfere with the kinetics and biodistribution of ^18^F-FES. It has been shown that rintodestrant (a selective ER degrader) affect ^18^F-FES kinetics altering both the qualitative and quantitative evaluation of FES-PET even if, 6 days after the end of treatment, the biodistribution returned to basal values as would be expected due to recovery of ER availability after drug washout [22].

FES-PET has the potential to become in the next future one of the most accurate imaging procedures to stage ER+ BC, especially for identifying MBC patients, and for evaluate the response to selective estrogen receptor degraders (SERDs).

## CANCER ASSOCIATED FIBROBLAST TARGETED POSITRON EMISSION TOMOGRAPHY RADIOPHARMACEUTICALS

Tumor microenvironment (TME) surrounding tumor cell is a complex and dynamic system playing a crucial role in the tumorigenesis and metastatic spread. The TME is composed of a huge number of different cell types that interact dynamically each other and with surrounding cancer cells. In BC, the most represented elements are the cancer-associated fibroblasts (CAFs) [23], that contribute to tumor cell invasion, migration, and growth, metabolic reprogramming, immunosuppression, and angiogenesis [23]. CAFs are characterized by the expression of a stromal antigen, the fibroblast activation protein (FAP), which is generally not observed in healthy tissues, but only in case of healing processes, remodeling, and inflammation [24].

Accordingly, quinoline-based inhibitors (FAPi) have been developed as radiopharmaceuticals [24] for exploring TME with PET imaging. The first reports showed higher diagnostic accuracy for FAPi-PET (sensitivity 100%, specificity 96%) compared to FDG-PET (sensitivity 78%; specificity 100%) in detecting primary tumor, lymph nodes, and distant metastasis. The main advantage was related to the more favorable biodistribution (no liver or brain physiological uptake), together with the ability to detect small lesions, even smaller than 5 mm [25,26]. These promising results have been confirmed in further studies with PET/MRI, that combine the added value of whole-body scanning and accurate morphologic imaging together with functional imaging of the TME [24]. The clinical experience of FAPi-PET in BC is limited at present, but this radiopharmaceutical has the potential to be a game-changer in BC in the next future.

Finally, FAPi can be labeled also with high energy isotopes for radio-ligand therapy, hence representing a theranostics agent. At present, only few evidence are available regarding the feasibility of ^177^lutetium-FAPi therapy in solid tumors and data derived from prospective clinical studies are needed to confirm the potential efficacy of FAPi in the treatment of MBC [27].

## HUMAN EPIDERMAL GROWTH RECEPTOR-2 TARGETED POSITRON EMISSION TOMOGRAPHY RADIOPHARMACEUTICALS

The HER2 is an orphan tyrosine kinase receptor member of the epidermal growth factor receptor (EGFR) family, involved in proliferation, migration, cell invasion and cell survival [28]. HER2 overexpression represents a negative prognostic factor [28]. However, its overexpression is a targeted therapeutic opportunity with the monoclonal antibody trastuzumab, a recombinant G1 immunoglobulin monoclonal antibody targeting the extracellular domain of HER2 [29^▪^]. Approximately 15% of BCs are defined HER2+ based on a combination of receptor overexpression (3+ or 2+) assessed by IHC and ERBB2 gene amplification assessed by *in situ* hybridization (ISH) [28,29^▪^,^30^]. Moreover, HER2 overexpression and amplification can present a heterogeneous pattern, and recent studies have shown that the presence of HER2 genetic heterogeneity is more frequent in cases with equivocal HER2 status by ISH and/or IHC [29^▪^]. Nevertheless, IHC is not exempt from false-negative or false-positive results due to inaccurate tissue handling (only 26% concordance among pathologists on scores 0 and 1+ [31^▪^]), or false-positive results due to fixation artifacts [32]. Indeed, along with the HER2+, there is HER2-low category (defined as IHC 1+ or IHC 2+ without HER2 gene amplification), comprehending approximately 50–55% of all primary BC [30,33^▪^]. Although most HER2-low BCs do not respond to trastuzumab, there is a subgroup of patients who could benefit from this therapy [33^▪^]. Furthermore, although trastuzumab therapy has been a milestone in the treatment of HER2+ BC, in recent years the number of approved anti-HER2 therapeutic agents has been significantly expanded leading to improved outcomes for patients with HER2+ and even HER2-low BC [34]. Therefore, identifying a noninvasive tool to monitor HER2 expression levels in vivo might contribute to improve the correct localization of HER2-positive tumor lesions, monitor the response to HER2-targeted therapy, and improve the selection of BC patients who may benefit from multiple antibody–drug conjugates. In this setting, the innovation is represented by the direct radio-labeling of the therapeutic agent.

Among different PET radiopharmaceuticals, [^89^Zr]trastuzumab and [^64^Cu]-DOTA-trastuzumab were those more extensively evaluated. Radiolabeled trastuzumab allows to locate HER2+ lesions considering the favorable TBR in the liver, spleen, kidneys and brain, supporting clinical decision-making when HER2 status cannot be established by biopsy [35]. [^89^Zr]trastuzumab uptake in the lesion is determined by multiple factors, including monoclonal antibody extravasation and interaction with the target. Moreover, among the semiquantitative PET parameters, SUV_max_ was higher in patients with BC HER2+ than in those with BC HER2−, but SUVmax was able to differentiate between IHC0 and IHC1–2–3 lesions only [36,37]. ^89^Zr has a long half-life (78.4 h) and therefore optimal images should be acquired 4 days after radiopharmaceutical administration [38]. Furthermore, this approach increases the radiation exposure of the patient [38]. This issue has been partially solved introducing Cupper-64 ([^64^Cu]DOTA-trastuzumab) having a half-life of 12.7 h. [^64^Cu]DOTA-trastuzumab demonstrated comparable radiation exposure to FDG-PET, due to low activity in healthy tissues despite high activity in blood pool [39]. However, high hepatic uptake was reported, thus affecting the accuracy in detecting small lesions [40]. This problem was partially solved by administering [^64^Cu]DOTA-trastuzumab after a standard therapeutic dose of trastuzumab (or after administering 45 mg of trastuzumab [41]) in order to decrease the liver uptake [40]. In preliminary studies [^64^Cu]DOTA-trastuzumab was able to distinguish HER2+ from HER2− lesions, as well as to monitor changes in HER2 expression after trastuzumab administration [39,40]. The radiolabeling of a monoclonal antibody used as therapeutic agent in clinical practice is an innovative approach, but further and extensive validation of this preliminary data is needed, to assess the efficacy of radiolabeled trastuzumab as PET tracer in clinical practice.

## INTEGRIN TARGETED POSITRON EMISSION TOMOGRAPHY RADIOPHARMACEUTICALS

Integrins are a family of heterodimeric transmembrane glycoproteins, promoting both cell-cell adhesion and cell−matrix interactions [42]. Recently, integrin alpha-vbeta-3 (α_v_β_3_) has been extensively investigated because of its expression by several tumor cells, as well as by endothelial and vascular smooth muscle cells in rapid division [42]. Receptors on the plasma membrane of BC integrin α_v_β_3_ (whose expression is quite heterogeneous [43]) have a role in modulating tumor cell proliferation, angiogenesis, local invasion, and metastatic spread. Integrin have been also proposed as prognostic parameter in BC [42]. Among different angiogenic signaling pathways, integrin α_v_β_3_ is implicated in the vascular endothelial growth factor (VEGF) pathway. The inhibition of VEGF significantly suppresses the expression of α_v_β_3_ on tumor cells resulting in the reduction of microvascular density, and so VEGF has been proposed as a potential biomarker of angiogenic activity [44]. This has increased the interest of molecular imaging with the exciting promise of a PET imaging of angiogenesis or tumor development, along with the knowledge that arginine-glycine-aspartic acid (RGD) peptide ligands have a high affinity for α_v_β_3_ integrin and can be radiolabeled [45]. This PET imaging would allow the selection of patients with BC who might benefit from antiangiogenic treatment, and to monitor the response to this therapy over time. [^18^F]galacto-RGD PET demonstrated to correctly measure in vivo the α_v_β_3_ expression [46]. Preliminary results also highlight a significant heterogeneity in α_v_β_3_ expression related to the predominant expression on endothelial cells alongside with tumor cells [47]. Unfortunately, [^18^F]galacto-RGD PET did not show optimal sensitivity for lymph-node staging [47]. However, promising results have been recently obtained with [^89^Zr]bevacizumab, which showed significantly higher uptake in tumor lesions than in healthy tissues, and it was observed that the replication rate was directly related to [^89^Zr]bevacizumab uptake [48]. Nevertheless, the sensitivity in detecting lymph node lesions remains suboptimal [48].

## CONCLUSION

PET imaging has the potential to illuminate different pathways in BC and gives an in vivo overview of specific tumor characteristics and tumor heterogeneity in a single-step examination. New generation imaging can improve the disease staging providing a more comprehensive overview of the tumor burden. FDG-PET might still play a role in dedicated setting, even if its efficacy in in low-FDG subtypes cannot be neglected, and new targets should be more extensively explored and validated. HER2 and integrin targeted PET radiotracers might provide useful information, namely selecting patients more likely to respond to a specific therapy. However, the level of evidence is low and definitive conclusion cannot be draws at present. FES-PET, as FDA-approved radiotracer, might be more extensively used to investigate ER+ patients, assess in vivo ER heterogeneity and for the early evaluation of SERDs efficacy. FAPi targets the TME and its role as diagnostic agent is still to be confirmed in BC but, as theranostic agent, its feasibility for radioligand therapy in MBC should be tested in further clinical studies. Finally, even if functional imaging represents in the next future an attracting opportunity for women affected by BC, the impact of new generation imaging on patient's survival is still to be confirmed.

## Acknowledgements

*None.*

### Financial support and sponsorship

*None.*

### Conflicts of interest

*There are no conflicts of interest.*

## REFERENCES AND RECOMMENDED READING

Papers of particular interest, published within the annual period of review, have been highlighted as:

▪ of special interest
▪▪ of outstanding interest
